# Navigating intersecting public health crises: a qualitative study of people with opioid use disorders' experiences during the COVID-19 pandemic

**DOI:** 10.1186/s13011-022-00449-3

**Published:** 2022-03-18

**Authors:** Dennis P. Watson, Monte D. Staton, Christine E. Grella, Christy K. Scott, Michael L. Dennis

**Affiliations:** 1 Chestnut Health Systems, 221 W. Walton St, Chicago, IL 60610 USA; 2 Department of Medicine, Center for Dissemination and Implementation Science, University of Illinois College of Medicine at Chicago, 818 S Wolcott Ave, Chicago, IL 60613 USA; 3 Chestnut Health Systems, 448 Wylie Dr, Normal, IL 61761 USA

**Keywords:** COVID-19, Medication for opioid use disorder, Methadone, Opioid use disorder, Pandemic, Recovery capital

## Abstract

**Background:**

The decades-long opioid epidemic and the more recent COVID-19 pandemic are two interacting events with significant public health impacts for people with opioid use disorder (OUD). Most published studies regarding the intersection of these two public health crises have focused on community, state, or national trends using pre-existing data. There is a need for complementary qualitative research aimed at identifying how people with opioid use disorder (OUD) are understanding, experiencing, and navigating this unprecedented time. The current study examines understandings and experiences of people with OUD while they have navigated these crises.

**Methods:**

The study was guided by a pragmatic lens. We conducted brief semi-structured qualitative interviews with 25 individuals in Chicago, the majority of which had received methadone treatment during the pandemic. Thematic inductive analysis was guided by primary interview questions.

**Results:**

The sample represents a high-risk group, being composed mostly of older non-Hispanic African American males and having considerable socioeconomic barriers. Themes demonstrate how individuals are keeping safe despite limited knowledge of COVID-19, how the pandemic has increased treatment motivation for some, how adaptations impacted treatment and recovery supports, how the availability social support had been reduced, and difficulties individuals had keeping or obtaining financial support.

**Conclusions:**

The findings can be useful for informing future public health response to ensure appropriate treatment access and supports are available. In particular are the need for treatment providers to ensure people with OUD receive appropriate and understandable health crisis-related information and ensuring funds are appropriately allocated to address mental health impacts of social isolation. Finally, there is a need for appropriate financial and infrastructure supports to ensure health and treatment access disparities are not exacerbated for those in greatest need.

## Background

Since its declaration in March of 2020, the COVID-19 pandemic has complicated and exacerbated efforts to quell the pre-existing and decades-long opioid epidemic. The most notable development attributed to the intersection of these two public health crises is a rise in overdose fatality [1–3], with the United States seeing nearly 100,000 overdose deaths over the first year of the COVID-19 pandemic, a more than 30% increase from the prior 12-month period [4]. To date, most research exploring issues related to the intersection of these two crises has investigated community, state, or national trends using pre-existing health system or state and federal mortality data [1, 2, 5–8], and this highlights a need for research aimed at understanding how people with opioid use disorder (OUD) are understanding, experiencing, and navigating this unprecedented time. Indeed, as the time of this writing, we are aware of only two published qualitative studies conducted in Canada that have sought to directly understand experiences of people with OUD during the COVID-19 pandemic [9, 10]. The current study addresses such issues using data collected in a US context through brief qualitative interviews with individuals living with OUD.

Several impacts of COVID-19 on individuals who use opioids have been highlighted in recent literature. A number of experts have postulated that people who use opioids and other drugs are at greater risk of COVID-19 complications than the general population [11–13], stemming from their higher rates of chronic health disorders associated with greater COVID-19 severity, and there is some evidence to support these individuals require more care and have higher vulnerability to death [14]. Regarding the previously mentioned overdose increase, factors that might underlie this trend that have been proposed include risky opioid use behaviors (e.g., using alone, changing dealers, increased use intensity), greater adulteration of the illicit drug supply, increased relapse risk, and disrupted access to treatment and services [3, 6, 15–17]. Additionally, several studies have provided evidence demonstrating overdose rates increased in a number of geographic areas following the enactment of COVID-19-realted shelter-in-places orders [1–3, 6]. There is also evidence that the burden of negative opioid-related outcomes has been unequally distributed during the COVID-19 pandemic, with overdose fatality rates rising among non-Hispanic African American males at almost twice the rate of their white counterparts [18].

Low-barrier, evidence-based treatment with sufficient planning and resources to support clients and ensure uninterrupted care is a necessary component to reduce the effects of the COVID-19 pandemic. The most effective approach to treating OUD is the long-term administration of any one of three medications [19, 20]: (1) methadone and (2) buprenorphine are both opioid agonists that occupy the brain’s primary opioid receptors and (3) naltrexone is an antagonist that completely blocks the same receptors. However, barriers to accessing and continued engagement in medications for OUD (MOUD) treatment are long-standing and numerous [21, 22], and these barriers can be compounded by unforeseen treatment disruptions during times of crisis [23]. In the case of COVID-19, shelter-in-place orders enacted early in the pandemic and the ongoing need for social distancing have conflicted with several federal rules requiring face-to-face interaction between providers and clients for buprenorphine and methadone treatment. Federal guidelines for opioid treatment programs are strictly enforced, stemming from historical concerns about diversion of the medications and require face-to-face examinations for buprenorphine prescribing and almost daily on-site dosing for most methadone clients [24]. In an attempt to ameliorate treatment disruptions caused by shelter-in-place orders, the U.S. Substance Abuse and Mental Health Services Administration adopted emergency guidelines allowing for telehealth prescribing of buprenorphine (which normally requires an in-person exam) and more flexible methadone take-home dosing (28- or 14-days depending on client stability) (see [25]). While viewed as highly beneficial, noted differences in implementation [26] likely resulted in varied effectiveness across organizations.

The current study expands on the research discussed above by investigating how individuals with OUD understood and navigated treatment and their personal recoveries during the COVID-19 pandemic. We undertook this work to inform our understanding of the MOUD treatment context that was impacting several of our current studies with protocols that had been designed prior to the pandemic’s start. Participant perspectives such as this are a recognized gap in the literature regarding OUD treatment changes during the COVID-19 pandemic [27].

## Method

This qualitative investigation was guided by a pragmatic paradigm. A pragmatic approach aligns with the primary goal of the study in that it views knowledge/beliefs, actions, and events as dynamic and contextually-bound [28–30]. This lens, as well as qualitative approaches in general [23, 31], can be useful for explaining such phenomena during times of rapid social change spurred by events such as a public health and social crisis. Further, pragmatic approaches favor the selection of sampling, data collection, and analytic approaches based on their ability to best acquire information suited to investigating issues of immediate concern—in this case the need to understand how COVID-19 was impacting the substance use treatment context given the lack of current research—rather than their fit with existing philosophical or methodological traditions [29, 30, 32].

### Sampling and recruitment

We employed a convenience sampling approach by recruiting participants from a list of individuals referred to MOUD treatment linkage through two projects based in Chicago, IL that were being run by members of the research team. This sampling frame included individuals referred to MOUD treatment either within the year prior to or after the start of the COVID-19 pandemic. Participants were informed of the study by a research staff member who was already working with them as part of one of the existing projects. Staff briefly described the study and asked prospective participants if they were interested. If interest was indicated, the staff person then either directly transferred them to the interviewer or arranged a time for the interview. For some who began treatment prior to the pandemic, we learned after the start of their interview that they had stopped treatment before the state’s shelter-in-place order went into effect. However, we decided to continue these interviews because the participants could still provide valuable information as to how their recovery had been impacted during the pandemic.

### Data collection

We developed the semi-structured interview guide to specifically understand the impact of the COVID-19 pandemic on interview participants’ treatment and recovery. The guide started by asking about each participant’s broad knowledge regarding COVID-19 to lay a foundation for understanding their experiences and reactions to the pandemic. We then followed with questions aimed at understanding how the pandemic had impacted the participant’s opioid use, treatment, and recovery from the start of Illinois’s shelter-in-place order on March 21, 2020  until the time of the interview.

Data collection occurred between September 29, 2020 and January 25, 2021. We determined data saturation to be obtained after 25 participants were enrolled because of redundancy of information learned from interviews at this point. This approach to saturation differs from that of theoretical saturation [33], and it is more useful in this instance given the pragmatic lens, finite list of potential interview recruits, and pointed nature of the interview guide. Due to social distancing protocols, interviews were completed over the phone. The first and second authors conducted interviews with separate participants. This two-interviewer strategy was employed because the population has inconsistent availability and phone access, making it difficult to schedule interviews in advance. Having two interviewers increased the chance that one would be available when a participant could be reached. Participants provided verbal consent. Interviews lasted between 7 and 25 min, with an average of 16 min. Participants received a $30 Visa gift card as an incentive.

### Data analysis

The first author led the data analysis with assistance from the second author, who are both trained qualitative researchers with experience conducting research on OUD treatment. The analysis focused on creating inductive codes using the primary research questions as an organizing structure [34]. The first author identified areas of text within the transcripts that related to specific questions asked during the interview (areas of the interview were ascribed to a question even if they were not specifically elicited by said question). He then developed inductive codes pertaining to the main questions, which were reviewed with the second author, and they then discussed areas of disagreement until full consensus was reached. The lead analyst then developed the broader themes in two ways: (1) identifying well-developed relationships among codes pertaining to specific questions and (2) identifying relationships among codes that cut across specific questions. Themes were reviewed with the other three authors, also experts in OUD treatment research, to help contextualize the data in light of pandemic-related treatment system changes of which they were aware.

## Results

Regarding participant characteristics, six identified as female and 19 as male, 24 identified as African American, and one identified as Hispanic/Latino. The mean age was 57 (range = 48–74 years). Data reflecting socioeconomic indicators were available for 24 participants^1^: mean education was 12 years (range = 10–14 years); 54% were living in independent housing, with the rest being doubled up in someone else’s housing (29%), unhoused (13%), or institutionally housed (4%); the majority were unemployed (54%) or disabled (33%); and median income was $637/month (range = $0-$4,500). All had past MOUD treatment experience; however, 20 had received treatment at some point after Illinois enacted its shelter-in-place order. Of these, 19 were receiving methadone treatment one was receiving buprenorphine; and 6 terminated treatment at some point between the start of the shelter-in-place order and the interview. All 5 participants who did not receive treatment after the enactment of the shelter-in-place order discontinued it prior to the pandemic’s official declaration; however, they were still able to discuss important issues related to their understandings of COVID-19 and its impact on their lives and recovery. Furthermore, 3 participants were in recovery or recovering from COVID-19 at the time of their interviews. We describe five themes that emerged from the analysis below.

### COVID-19 knowledge and related prevention

Television or internet-based news was the primary source of COVID-19 information discussed, with participants making statements such as “I only know what they say over the TV” (51-year-old female). Other sources included “word of mouth” (62-year-old male), such as in interactions with family, friends, or other people in the community. Some individuals discussed receiving information from healthcare providers; however, only two specifically discussed their MOUD provider as a source of COVID-19 information: “They [the methadone clinic] got it posted on the wall, [they] talk to you about it when you first come in [for treatment]” (53-year-old male).

Most statements reflected how participants understood the disease to be serious and deadly: “I know it’s killing people. I know it’s a very bad virus, and it’s rough. It’s got people scared.” (53-year-old male). A handful of participants framed their discussion of the virus from a risk perspective, stating that older individuals and people with preexisting conditions, particularly respiratory/breathing issues, were at greater or the only ones at risk:

I know the virus, most times people that do have problems with it, it’s because they have respiratory problems in the first place. You know, they have something wrong with their, they lungs or with their breathing. It’s not just something that anybody can catch, [it’s] just when you already have some kind of [health] problems already in your life. (67-year-old male)

In addition to showing this participant’s correct understanding of the higher risk associated with respiratory problems, the above quote represents a frequent misunderstanding identified across interviews that only those with pre-existing conditions or who are older are at risk of catching the virus. This same participant went on to speculate whether opioid use might be linked with COVID-19 risk: “…would it [using opioids] increase your likelihood of you becoming ill [with COVID-19]?”.

Discussions also demonstrated participants were using their knowledge of the virus to reduce risks associated with it. Indeed, all participants discussed taking preventative measures to keep themselves safe:

Keep your hand washed and sanitized, you have to keep a safe distance between those people, social distancing. Also, the mask. Keep your hands washed and disinfected and try not to be in crowds. And if you have any symptoms, fevers, headaches, then know something’s probably going wrong. So, you gotta watch for those things as well. (59-year-old male)

While this person gave a detailed account of precautions people can take, most other participants limited their discussions of precautions they were taking to mask wearing and social distancing.

### Pandemic increases treatment motivation

A number of participants discussed how pandemic-related factors contributed to their decision to start and/or remain in treatment. In some cases, participants’ motivation to begin treatment during the pandemic stemmed directly from their inability to secure a stable income that could support their opioid use:

It [the pandemic] stopped me being able to do what I needed to do. Because [there are] not many people on the street no more [to panhandle from]. [I] can’t get no job to help me out. I couldn’t get what I need [money to purchase opioids] because people don’t want to deal with people, not much work right now. It affected me a whole lot…[I] can’t get nothing on the streets, it’s too hard now. (53-year-old male)

A different participant expressed a similar sentiment: “I was getting enough [opioids] to take care of myself every day [before the pandemic]…[If] I could get what I need[ed] for self-medication, then why would I need to go to a program?” (74-year-old male). This statement demonstrates how for some individuals obtaining a regular supply of opioids is a form of self-medication that prevents them from experiencing opioid withdrawal symptoms, rather than just a way to get high. The participants who stated to two prior quotes explicitly identified that their inability to procure monies through panhandling was their primary barrier to obtaining illicit opioids.

Another motivation for starting or staying in treatment was the need for social distancing during the pandemic. Socializing is a means for obtaining opioids, and “when you’re not going out [as] much as you used to and you’re not socializing like you used to, you[‘re] not getting on your habit [i.e., able to use opioids]” (57-year-old male). Another participant stated “it has been a big help, the methadone…because I don’t have to go search for drugs” (53-year-old female), and searching for drugs was the only form of social interaction she was having prior to treatment since she had lost her factory job and could not visit family due to travel restrictions.

Finally, one participant was motivated to start treatment because the illicit opioid supply had become increasingly adulterated since the start of the pandemic, stating: “They put so many other things [in it], the heroin is not just heroin anymore” (49-year-old female). From her perspective, treatment was a means of protection from a life-threatening situation that could result from adulterated opioid use.

### The changing nature of interactions with treatment and recovery supports

Participants discussed both positive and negative interactions with treatment and recovery support services. These discussions were reflected in the adaptations providers had made to accommodate shelter-in-place orders and social distancing. Participants who were in methadone treatment (which was the majority) discussed how changes in regulations allowed more frequent take-home doses so they no longer had to report to the clinic on a daily basis: “[Before the pandemic] we would go every day [to the clinic]…now, you go two days out of the week. You just get your doses that you supposed to drink for that day, and they give you bottles to take home with you” (63-year-old male). Another participant described a similar dosing model, as well as their preference for it:

You basically show up two days out of the week to get that day’s [dose] and pick up bottles, you have to have your clean drop, you can take bottles home…I prefer twice a week personally because I have other things [to do], and like I say, all addicts are not the same. (49-year-old male)

The final part of this quote refers to the participant’s view that some methadone clients are more responsible than others and therefore can be trusted to receive more take-home doses. While these participants described having received 2–3 days’ worth of medication at a time, others reported receiving as much as 30 days.

Not having to go to the clinic daily was seen as a benefit to some; however, additional adaptations made to accommodate social distancing had troubling impacts. One such example was reduced staffing and the elimination of in-office services:

The only people that be there [at the methadone clinic] is the people that, the security that take your temperature and give you a mask before you go in, and the people that’s inside the building they sitting back behind a big old desk with plastic all around it. And, it’s just really hectic, you can’t communicate with people. They can’t hear you, you keep on saying the same thing two or three times. You can’t actually go up to talk to someone to find out something. It’s really ridiculous. And, if you do get an understanding from someone behind the desk, then you gotta go call the person. You can’t go see them…it’s just made things terrible. (67-year-old male)

Other participants lamented the elimination of in-person individual and group counseling sessions that came with the move to telehealth. One participant even viewed social distancing adaptations as callousness on the part of staff: “They don’t want to deal with you because the epidemic doing that. They don’t want to help you out, they don’t want to be close to you” (59-year-old male).

### Reduced social support

Participants provided a number of examples showing how the availability of social support had dwindled during the pandemic. Participants were seeing their family and friends less often due to social distancing needs. When asked how the pandemic was affecting her recovery, one participant stated: “Just, right now it’s [a main part of recovery is] going to see my daughter. She’s in [name of city], and, actually, I’m not able to go back and forth to see her either because of this pandemic…and my daughter that’s in the military, she can’t come home from Germany because they under lock [down] too” (53-year-old female). Other participants spoke about not being able to visit with children and grandchildren as frequently or at all. The inability to socialize with friends or even having had close friends who died due to COVID-19 were additional concerns expressed. One participant who had four friends infected with COVID-19 stated: “…one of the guys that died, I was pretty close to, so emotionally, that hurt a lot” (52-year-old male).

Peers who are also in recovery provide a source of social support that some participants rely heavily on and were unable to access. An important source of peer support one participant greatly missed was her 12-step meetings:

…when it [the pandemic] first came about, we were not having meetings, we were not allowed to go to meetings because they had shut all of that down…Well, actually, it’s made me lazy. Not getting up and making meetings begins to make you become more idle. And then you become complacent and then it’s easier for you to pick up again…without the fellowship, you know, it’s hard to stay, it’s hard to stay focused and stay clean. Not to say that we can’t stay clean on our own, but it’s just, the difference about being around people, seeing how people are living their lives on a day-to-day basis without using, that is like a big motivation for me. (57-year-old female)

In a similar vein, another participant discussed how the elimination of social events usually facilitated by their treatment provider was negatively impacting them:

…it has affected my life as far as social distance, going to social events and things of that nature…you can’t attend any social events because they’re not having any. They [i.e., the treatment provider] used to have parties or social, sober gatherings. You can’t go anywhere. You just sit around bored all day. (63-year-old male)

### Inability to find financial support

Participants discussed considerable difficulties keeping or obtaining financial support. As already discussed above, some individuals described how the pandemic had negatively affected their ability to panhandle due to reduced foot traffic during shelter-in-place orders or people taking social distancing precautions. Even more frequently discussed was participants’ inability to keep employment:

Well, actually, I was doing warehouse work…I was only doing it two more years [before I could] sit down and retire…They laid people off because, actually, the plant was shut down for a while. It was because of the COVID. (57-year-old male)

This is demonstrative of a common employment issue faced by several participants, being laid off or let go of their jobs due to the pandemic. For those who were laid off, it was unclear when or if their employers would ever bring them back to work.

Those participants who were looking for work discussed how the pandemic was making their employment search challenging. While some individuals discussed how employers were not hiring because of the pandemic, others discussed how the application process itself had become more difficult:

No, cause some places, they tell you to go online and fill out the application much more now. And back before the pandemic, you could go into the office and sign out the application and they basically tell you if you going to get the job or not get the job. But [completing employment applications on] the computer, I think is a long period to wait [to hear anything back]. (58-year-old male)

Because of social distancing, more employers were requiring people to fill out online applications, which was perceived to elongate the entire application process and/or significantly reduce people’s chances of getting hired.

## Discussion

Our findings address a need for more research regarding the personal impact of the COVID-19 pandemic on individuals with OUD and how they have navigated MOUD treatment and recovery during such a chaotic time. While we spoke with 6 women and one Hispanic male, the themes mostly reflect the experiences of older non-Hispanic African American males, a high-risk group that has experienced a rise in opioid overdose-related fatality during the pandemic that is unmatched by any other demographic group [18]. Additionally, the majority of those with whom we spoke had low education, high rates of unemployment, and low income, and half were not independently housed. All of these factors are noted social determinants that can structure opioid and other substance users’ risk environments toward more negative health outcomes [35–37], making the findings relevant to understanding the impact of the COVID-19 pandemic on other highly marginalized opioid users [38]. Four of the themes identified align with similarly-focused qualitative work conducted in Canada that have demonstrated varying levels of COVID-19 understanding and protective efforts [9], negative experiences related to reduced MOUD treatment capacity and care quality due to pandemic-related regulations [9, 10], diminished social support [9], difficulty obtaining financial support [9], and disruptions in general medical services and food access [10]. The fifth theme, which is unique in relation to previously cited qualitative research and has not been explicitly discussed in prior work, is a link between the pandemic and treatment motivation. We discuss the implication of these findings as they relate to the wider field of literature below.

There is a need for more research to investigate the perceptions of people with OUD as they relate to understandings of COVID-19, exposure risk, and preventative actions. Despite admittedly limited COVID-19 knowledge, the individuals we spoke with were generally trying to follow public health guidance. Yet the interviews revealed there was still some misinformation regarding COVID-19 contraction risk, such as the belief that those without preexisting respiratory issues could not catch the virus. It is concerning that such misinformation exists within the sample since targeted and appropriate education is necessary during times of health crisis [39], particularly among a group with potentially greater risk of negative health outcomes [14]. Additionally, poor information from public health authorities is a noted stressor during quarantines [40], and stress can precipitate relapse [41]. There is also precedent to suggest improved education could reduce both COVID-19 risk and associated substance use, as one recent study found smokers and vapers who understood their heightened risk of COVID-19 infection or complications used this as a motivation to quit [42].

The motivation to start treatment was a positive consequence of the pandemic discussed by some interview participants. Interruptions in the illicit opioid supply can lead individuals to change drug use behaviors for a variety of reasons including the desire to stop withdrawal symptoms and potential increase in overdose risk. This is supported by a qualitative study of Canadians who use drugs (including opioids) conducted by Ali et al. [43], which found interview participants attributed changes in substance use behavior during the current pandemic to supply interruptions and increased overdose risk. These findings and our own also point to fear of COVID-19 exposure as another motivating factor for behavior change related to drug use. As such, drug supply interruptions and fear of potential COVID-19 exposure presented opportunities to engage individuals in treatment. However, it is unlikely this opportunity was fully seized in the initial weeks of the pandemic because providers were trying to solve the problem of how to serve clients in the midst of shelter-in-place orders and to implement loosened telehealth and dosing guidelines [26, 44]. Furthermore, the lack of OUD-specific COVID-19 risk knowledge discussed above points to the potential for risk education to improve treatment uptake for people with OUD.

The guidelines that loosened take-home dosing and telehealth regulations were implemented to improve MOUD access and continuity, which is difficult during a societal crisis [23]. Our participants largely viewed relaxed dosing guidelines as beneficial, which is not surprising considering take-home restrictions are an identified barrier to methadone uptake and allowing patients more frequent take-home doses early in treatment can increase retention [45, 46]. However, social distancing-based service changes and reductions to on-site services were discussed as negatively impacting the perceived quality of treatment and recovery supports (e.g., in-person counseling, 12-step, provider-organized social events) for some individuals with whom we spoke. This is concerning, as reduced services are a driver of premature treatment discontinuation and relapse risk [15, 47]. Some participants greatly missed the availability and interactions with counselors. While new guidelines meant these interactions could shift to a telehealth format, implementation of telehealth services during the pandemic has been inconsistent [48], resulting in barriers to these services for many who need them [49].

Recovery capital refers to the resources people have available to put toward establishing and maintaining their recovery [50], and two key areas of recovery capital, social support and employment, were negatively impacted by the pandemic. Shelter-in-place orders had negative impacts on social support in our sample, and experts in the treatment community have commented regarding their concerns that such social isolation can increase relapse risk [51], as well as greater risk of overdose fatality if using alone, without peers who can intervene if needed [52, 53]. Positive social connections can motivate people to engage with treatment and improve associated outcomes [54], and previous research has demonstrated how disruptions in such connections can negatively impact an individual’s well-being in times of forced social isolation [40, 55]. Investing in the expansion of peer support services (e.g., paraprofessionals with lived experience of recovery who can provide support and motivation) has been a recommended approach in one expert commentary for mitigating some of the problems of forced isolation that could lead to mental distress and substance use [56]. Indeed, there is documentation of organizations increasing peer support check-ins through telehealth early in the pandemic [57]. The ability to quickly mobilize such supports could be beneficial in future times of crisis. Peer support specialists could also be used to assist people with employment issues, the other key aspect of recovery capital discussed. Employment is key to recovery in that it provides meaning to people’s lives, as well as insurance and income that can help cover treatment costs and improve retention [58].

Regarding limitations, as previously discussed, older non-Hispanic African American males receiving methadone treatment make up most of the sample. While this limits the degree to which our findings might be applicable to other groups (e.g., women, other racial, ethnic, or cultural groups, or those receiving different MOUDs) in other locations, it is important to consider the goal of inductive qualitative research is often to link phenomena of interest to the context of focus and to strive for theoretical (rather than statistical) generalizability [59, 60]. From this perspective, the sample is a strength given it reflects the population most impacted by the opioid epidemic in Chicago [61]. Moreover, the themes both align with the limited qualitative research on the topic completed to date while also providing some new theoretical insight into the impacts of the pandemic on a high-risk group of opioid users. The quality of the data was likely limited by the phone interview approach used to ensure social distancing. The duration of the calls was shorter than expected because participants were largely anxious and often expressed a strong desire to end the calls sooner than the 45 to 60 min for which they were scheduled. Some of the reasons for this included: (a) participants sharing a phone with others who needed to use it, (b) having others in the room who were urging them to finish; and (c) participants scheduling calls when they were engaged in other activities (e.g., traveling in the car, waiting for an appointment). The interviewers were not able to overcome these competing demands through rephrasing questions or probing despite having more than a decade of qualitative interviewing experience each. Because of their knowledge of the population, the interviewers expected difficulties conducting interviews over the phone. While not optimal, this limitation was unavoidable given the need for social distancing that prevented in-person interviews. Illinois’s shelter-in-place order (effective from March 21, 2020 through May 29, 2020) ended prior to the start of data collection, and this could have impacted the quality of data related to participants’ ability to recall their earlier experience under this order. However, many of the changes made to treatment that had occurred in response to this order have remained in effect out of the need for continued social distancing, particularly during the subsequent variant-related surges. Reaching informational saturation suggests the data provided a representative picture of participants’ experiences, and it is likely the targeted nature of the interviews and our decision to focus on data/information redundancy (rather than theoretical redundancy) allowed us to reach saturation despite the brevity of interviews [33].

## Conclusions

This is one of the few studies to date that has sought to understand the experiences of people with OUD during the COVID-19 pandemic. While the study’s primary goal was to inform our existing research, the questions guiding our pragmatic design were useful for identifying a number of areas for possible responses to future OUD treatment and recovery support disruptions [31]. In particular, our findings demonstrate a need for treatment providers to ensure people with OUD receive appropriate health information in situations where they are at greater risk of transmission or complications of a particular disease. There is also a need to ensure funds are appropriately allocated to address the effects of social isolation some have commented could precipitate relapse [51], such as increased funding for the expansion of peer support services. Although revised dosing and telehealth guidelines have been helpful, the struggle to implement them resulted in missed opportunities to engage people who might have been looking for treatment early in the pandemic due to initial drug supply interruptions. Keeping these guidelines in place would serve as a foundation for longer-term changes that make MOUD more accessible and result in a nimbler system that could respond faster at the start of the next public health crisis [25, 48, 62]. Finally, the sample’s generally low socioeconomic status and inability to obtain stable employment experienced by some participants drives home the relationship between poverty and health—particularly among individuals with substance use disorders—that needs to be considered when developing a crisis response. Overlooking this relationship ignores the contextual realities of people with OUD, which can result in well-meaning policies with serious unintended consequences for marginalized groups, such as the contribution of 2020’s shelter-in-place orders and the continued need for social distancing to the rise in overdoses demonstrated in some areas [1–3, 6].

## Data Availability

Qualitative data are not available due to confidentiality concerns related to such a small sample.

## Abbreviations

OUD: Opioid use disorder
MOUD: Medications for opioid use disorder

## Footnotes

1 These data came from the research projects from which participants were sampled, which was missing for one participant.
